# Papillary glioneuronal tumors: histological and molecular characteristics and diagnostic value of SLC44A1-PRKCA fusion

**DOI:** 10.1186/s40478-015-0264-5

**Published:** 2015-12-15

**Authors:** Melanie Pages, Ludovic Lacroix, Arnault Tauziede-Espariat, David Castel, Estelle Daudigeos-Dubus, Vita Ridola, Sophie Gilles, Frederic Fina, Felipe Andreiuolo, Marc Polivka, Emmanuele Lechapt-Zalcman, Stephanie Puget, Nathalie Boddaert, Xiao-qiong Liu, Julia A. Bridge, Jacques Grill, Fabrice Chretien, Pascale Varlet

**Affiliations:** Department of Neuropathology, Sainte-Anne Hospital, Paris, France; Paris Descartes University, Sorbonne Paris Cité, Paris, France; Institut National de la Sante et de la Recherche Medicale, INSERM CEAUnit1000, “Imaging & Psychiatry”, University Paris Sud, 91400 Orsay, France; Gustave Roussy, Département de Biologie et Pathologie Médicales, Villejuif, F-94805 France; CNRS, UMR8203, Villejuif, F-94805 France; Univ Paris-Sud, UMR8203, Villejuif, F-94805 France; Gustave Roussy, UMR8203, Villejuif, F-94805 France; Gustave Roussy, Département de Cancérologie de l’Enfant et de l’Adolescent, Villejuif, F-94805 France; Department of Pediatric Hematology and Oncology, Catholic University, Rome, Italy; Service de transfert d’Oncologie Biologique, LBM APHM, Marseille, France; Department of Pathology, Lariboisière Hospital, Paris, France; Laboratoire d’Anatomie Pathologique, CHU de Caen, France, UMR6301-ISTCT CNRS, CEA, Université de Caen Basse-Normandie, GIP, Cyceron, France; Department of Pediatric Neurosurgery, Necker Enfants Malades Hospital, Paris, France; Department of Pediatric Neuroradiology, Necker Enfants Malades Hospital, Paris, France; Department of Pathology and Microbiology, University of Nebraska Medical center, Omaha, NE USA; Department of Pediatrics, University of Nebraska Medical Center, Omaha, NE USA; Department of Orthopaedic Surgery, University of Nebraska Medical Center, Omaha, NE USA; Institut Pasteur, Human Histopathology and Animal Models Unit, Infection and Epidemiology Department, Paris, France

**Keywords:** Papillary glioneuronal tumor, *SLC44A1-PRKCA*, MAPK, *BRAF*, *FGFR1*, *KIAA-BRAF*, *rosette-forming glioneuronal tumors of the fourth ventricle*, *ganglioglioma*, *angiocentric neuroepithelial tumors*

## Abstract

**Introduction:**

Papillary Glioneuronal Tumor (PGNT) is a grade I tumor which was classified as a separate entity in the World Health Organization Classification of the Central Nervous System 2007 in the group of mixed glioneuronal tumors. This tumor is rare and subclassifying PGNT represents a challenge. Recently, a fusion between *SLC44A1* and *PRKCA* which encodes a protein kinase C involved in MAPK signaling pathway has been described in two studies (five cases). The current study aimed at raising the cytogenetic, histological and molecular profiles of PGNT and to determine if *SLC44A1-PRKCA* fusion represented a specific diagnostic marker to distinguish it from other glioneuronal tumors.

**Results:**

We report on four pediatric cases of PGNT, along with clinico-radiologic and immunohistological features for which *SLC44A1-PRKCA* fusion assessment by fluorescence in situ hybridization, *BRAF V600E* and *FGFR1* mutation by immunohistochemistry and direct DNA sequencing and *KIAA1549-BRAF* fusion by RT-PCR were performed. MAPK signaling pathway activation was investigated using phospho-ERK immunohistochemistry and western blot. We analyzed fifteen cases of tumors with challenging histological or clinical differential diagnoses showing respectively a papillary architecture or periventricular location (PGNT mimics). fluorescence in situ hybridization analysis revealed a constant *SLC44A1-PRKCA* fusion signal in all PGNTs. None of PGNT mimics showed the *SLC44A1-PRKCA* fusion signal pattern. All PGNTs were negative for *BRAF* V600E and *FGFR1* mutation, and *KIAA1549-BRAF* fusion. Phospho-ERK analysis provides arguments for the activation of the MAPK signaling pathway in these tumors.

**Conclusions:**

Here we confirmed and extended the molecular data on PGNT. These results suggest that PGNT belong to low grade glioma with MAPK signaling pathway deregulation. *SLC44A1*-*PRKCA* fusion seems to be a specific characteristic of PGNT with a high diagnostic value and detectable by FISH.

## Introduction

Mixed neuronal-glial tumors, a category in the World Health Organization (WHO) Classification of the Central Nervous System, are heterogeneous and composed of variably differentiated neuronal and glial cells.

Papillary Glioneuronal Tumor (PGNT) is a grade I glioneuronal tumor which was first described by Komori and colleagues in 1998 [1]. Recognized in WHO 2000 classification as a variant of ganglioglioma, PGNT was classified as a separate entity in WHO 2007 classification [2].

This tumor is rare, with approximately 70 cases reported in the last decade [3, 4]. Classifying PGNT is a challenge with a diagnosis generally based on neuroimaging and histology. However, the magnetic resonance imaging (MRI) characteristics are not specific, highlighting well-demarcated lesions usually near the ventricles, and are rather used to search for arguments against the PGNT-mimics tumors as IV ventricle, pineal or intracortical locations. Histologically, PGNT presents a biphasic and biphenotypic differentiation and is characterized by a glial component arranged in papillary architecture overlaying hyalinized vessels, associated with interpapillary regions containing homogeneous oligodendrocyte-like, neurocyte-like cells, ganglioid-cells and ganglion cells [2].

Studies examining genetic alterations in PGNT are few. Their molecular characteristics are not yet completely clear. Gain and structural abnormalities of chromosome 7, isolated or among other abnormalities have been described. However, no *EGFR* gene amplification has been observed [5, 6]. Fusion genes or mutations involving *BRAF, FGFR1* and the MAPK pathway have been described in other glioneuronal or glial tumors such as ganglioglioma or pilocytic astrocytoma [7–10]. *BRAF* mutation has been analyzed in only two cases, which were negative [6]. A single case report described a *FGFR1* mutation by pyrosequencing (*FGFR1* N546K) but no large pediatric low grade gliomas (pLGG) cohort studying *FGFR1* mutational status have included some PGNT investigating *FGFR1* mutation in PGNT [11]. Yet, to our knowledge, *KIAA*-*BRAF* fusion has not been studied in PGNT. Bridge and colleagues identified a recurrent chromosomal translocation t(9;17)(q31;q24), with a resultant oncogenic fusion protein SLC44A1-PRKCA, in three PGNTs. This fusion is detectable by conventional cytogenetic analysis and fluorescence in situ hybridization (FISH) [12]. A recent study has confirmed the presence of the fusion in two additional PGNTs [13].

In the current study, we investigated four pediatric cases of PGNT, along with clinico-radiologic, follow-up and immunohistological features, including *BRAF* (mutation and fusion) and *FGFR1* status, for the *SLC44A1*-*PRKCA* fusion by FISH analysis. In addition, in order to demonstrate MAPK pathway activation, we analyzed phospho-ERK expression by IHC and western blot. Moreover, we analyzed fifteen cases of rare tumors either showing a papillary architecture or presenting within the clinico-radiological differential diagnosis of pediatric neuro-oncology (PGNT mimics).

## Materials and methods

### Tumor samples

The study was carried out on four cases classified as PGNT at the time of initial diagnosis. Likewise, two gangliogliomas with papillary architecture, two ANETs, five RGNTs, two PRPs, one PE, two neurocytomas and one astroblastoma were collected for this study (PGNT mimics). With the exception of one case from Lariboisière Hospital, all cases were retrieved from the pathology archives of Sainte-Anne-Necker Hospital and were subject to a local histopathological review (PV). Sections for genetic analyses and immunohistochemistry were prepared from zinc formalin-fixed paraffin-embedded tissue specimens (formalin 5 %, zinc 3 g/L, sodium chloride 8 g/L).

### Immunohistochemistry

Immunostaining was performed at the time of initial diagnosis. Representative zinc formalin-fixed sections were deparaffinized and were subject to a Ventana autostainer (BenchMark XT, Ventana Medical Systems or Discovery XT, Ventana Medical Systems) according to standard protocol. The following primary antibodies were used: Glial Fibrillary Acidic Protein (GFAP) (1:200, 6 F2, Dako Denmark A/S, Glostrup, Denmark), synaptophysin (1:20, SY38, Progen Biotechnik GmbH, Heidelberg, Germany), CD34 (1:40, QBEnd-10, Dako, Denmark A/S, Glostrup, Denmark), chromogranin A (1:200, LK2H10, Diagnostic BioSystems, Pleasanton, USA), phospho-FGFR1 (Y653/654) (1:75, PA5-12594, Thermoscientific, Waltham, USA), p53 (1:5000, DO-1, Santa Cruz Biotechnology, Dallas, USA), *BRAF* V600E (1:100, VE1, Spring Bioscience, Pleasanton, USA), histone H3.3 K27M mutation (1:1000, ABE419, EMD Millipore, Billercia, USA). The chromogen diaminobenzidine was employed. Slide scanning was performed using NanoZoomer 2.ORS (Hamamatsu photonics, Hamamatsu, Japan).

### SLC44A1-PRKCA FISH analysis

Molecular cytogenetic (FISH) analysis was performed on representative tumor sections (4 μm) part as described by Bridge and colleagues [12] using prelabeled (5-TAMRA or 5-fluorescein-deoxyuridine triphosphate) bacterial artificial chromosome (BAC) probes (Empire Genomics, Buffalo, NY), covering *SLC44A1* on 9q31 region (RP11-24 J9, RP11-1097P14, RP11-95O7, RP11-235C23) and *PRKCA* on 17q24 region (RP11-98C3, RP11-188A11, RP11-1036I14, RP11-51D14, RP11-52B5). The genomic location of each BAC set was verified by hybridization to metaphase chromosomes of normal peripheral blood lymphocytes.

FISH study was performed on interphase nuclei following standard procedures. Briefly, four-micron sections of tumor were mounted on SuperFrost Plus slides (Erie Scientific CA., Portsmouth, NH) and the area to be probed was determined in accordance with hematoxylin and eosin stained section. The sections were deparaffinised in xylene, rehydrated through an ethanol series air-dried and incubated in pre-treatment solution (1 M NaSCN-tris) at 80 °C for 25 min. Slides were then treated with a 0.01 % pepsin solution (Sigma-Aldrich, Saint Louis, USA) at 37 °C for 10 min. After dehydration, 10 μl of probe mixture was applied to each sample, slides were coverslipped and codenatured at 73 °C for 2 min and hybridized at 37 °C for 24 h using thermobrite system (Leica Biosystems, Richmond, IL). A post-hybridization wash was performed in 2xSSC at 73 °C for 2 min. Preparations were dehydrated and counterstained with 4,6-diamidino-phenyl-indole (DAPI). Signals were scored in at least 100 non-overlapping interphase nuclei. A negative control was included (brain parenchyma in tumor periphery). Positive control consisted of three cases of PGNT confirmed by Bridge and colleagues as described in the original article [12]. Specimens were considered *SLC44A1-PRKCA* positive if signal fusion(s) were detected in more than 20 % of nuclei analyzed. Results were recorded using a DM600 imaging fluorescence microscope (Leica Biosystems, Richmond, IL) fitted with appropriate filters, a CCD camera, and digital imaging software from Leica (Cytovision, v7.4).

### BRAF molecular analysis

DNA was extracted from 20-μm-section paraffin embedded tumor samples after a 24 h digestion by proteinase K, using the DNeasy Tissue Kit and the QIAcube automated extractor (Qiagen, Hilden, Germany). Yield and quality of DNA were evaluated by Qubit fluorometer (Invitrogen, Carlsbad, CA). Direct Sanger sequencing for mutational assessment of exon 15 of *BRAF* was performed following PCR amplification as previously described [14]. PCR was carried out on 20 ng of DNA in 10 μl final volume and 1 U of Hot Start Taq polymerase (Qiagen). The amplified products were studied by direct sequencing after clean-up exonuclease ExoSAP-IT (Affymetrix, Santa Clara, CA) using the Big Dye Terminator Cycle Sequencing Kit and capillary electrophoresis on the automated sequencer ABI3730 (Applied Biosystems, Carlsbad, CA). Sense and antisense sequences were screened for exonic alterations using SeqScape v2.5 software (Applied Biosystems) and compared with the NCBI reference sequences: *BRAF* (NM_004333.4).

### KIAA1549-BRAF qRT-PCR analysis

RNA was purified from tissue section with RNeasy Micro Kit (Qiagen) according to the manufacturer’s recommendations. Hydrolysis probe assays to detect K-B exon junctions were designed according to Tian and colleagues and purchased from Life Technologies (Darmstradt, Germany) [15]. Glyceraldehyde-3-phosphate dehydrogenase (GAPDH) mRNA was used as internal control. PCR amplification was carried out on cDNA from 10 ng of RNA using AmpliTaq Gold DNA polymerase (Life Technologies). Reactions were in duplicate with incubation at 50 °C for 2 min, then 95 °C for 10 min, and then for 50 cycles of 95 °C for 15 s and 60 °C for 1 min. Fluorescence was recorded and cycles to threshold (CT) are calculated using ViiA7 RUO Software (Life Technologies). A reference of total RNA obtained from non-neoplastic cerebellum tissue sample (Stratagene, LA Jolla, CA) was used as negative control. The positive control for *KIAA1549-BRAF* was RNA with known breakdown junction, which was provided by Dr Jones in Heidelberg.

### FGFR1 molecular analysis

We extracted DNA from 4 μm formalin fixed paraffin embedded tumor sections blades. Each blade was accompanied by a blade coloured with Hématoxyline-Eosine-Safran (HES) on which the zone containing the tumorous cells was emphasized by strapping and the proportion of tumorous cells indicated. The first stage consisted in dewaxing by Microclearing® (DiaPath, Italy) followed by slow tissue rehydration. DNA were purified by automated extraction on EVO75 (Tecan), according to the Macherey-Nagel protocol; NR (NucleoSpin® 96 Blood; NucleoSpin® 8 Viruses Binding Strips; Macherey Nagel). We used qPCR-High Resolution Melting (qPCR-HRM) for *FGFR1* exon 12 and 14 as a screening technique, allowing avoiding useless sequencing of not mutated samples [16]. We used touchdown PCR conditions with one step 95 °C 10 min and 45 cycles (95 °C 10 s, 65 °C to 58 °C (0.5 °C); 72 °C 30 s). Classification is determined by sequencing Sanger [17], (MixBigDye® 5X, Roche) after DNA purification (ExoSap-IT®), pipeting being automated on Evo75®, (Tecan). The sequences are analyzed on 3500 or 3130 Dx Genetic Analyser® (Applied Biosystems). The analytical chain was accredited according to Iso-Norm 15189 (agreement num: 8-1739) [18, 19].

### Phospho-ERK analysis

Immunostaining was performed as described above, using phospho-ERK1/2 antibody (Thr202/Tyr204) (1:800, D13.14.4E, Cell signaling, Danvers, USA). For the western blot analysis, total tumor lysates were generated as previously described [20]. Thirty micrograms of protein per sample were separated electrophoretically in 4–15 % precast SDS polyacrylamide gels and transferred to precut nitrocellulose membranes using the Trans-Blot® turbo™ Transfer Starter System (all Bio-Rad). Chemiluminescence and colorimetric detection were performed using ChemiDoc™ MP Imaging System and horseradish peroxidase conjugated rabbit polyclonal anti-human p-ERK1/2 (Thr202/Tyr204), ERK1/2 (1:1000) detected with peroxidase-conjugated secondary anti-rabbit antibody, respectively (1:1000; Cell Signaling Technology) and HRP conjugated mouse monoclonal antibody anti-human β-Actin (S125; all 1:1000), followed by chemiluminescence solution (Clarity™ Western Chemiluminescent HRP Substrate; Bio-Rad).

## Results

### Clinical and neuroradiological features

Characteristics of the patients and controls are respectively summarized in Tables 1 and 2.

**Table 1:** Summary of PGNT cases: clinical and radiological characteristics and follow-up

	Sex	Age (y)	Location	Cyst	GTR	Clinical follow-up	Status at follow-up
case 1	F	6	parietal	yes	yes	radiotherapy, second location temporal treated by surgery	NED (6 y)
case 2	M	8	intraventricular (V3)	no	yes	-	NED (9 y)
case 3	M	14	temporal	yes	yes	residual micronodule	non progressive (3 y)
case 4	F	5	parietal	no	no	-	NED (9 m)

*F* female, *GTR* gross total resection, *M* male, *m* month, *NA* not available, *NED* no evidence of disease, *y* year

**Table 2:** Summary of basic clinical characteristics of PGNT mimics cases

	Sex	Age (y)	Location	Diagnosis
case 5	M	4	chiasmatic	ganglioglioma with papillary architecture
case 6	M	42	frontal	ganglioglioma with papillary architecture
case 7	F	8	temporal + thalamopeduncular + chiasmatic	ANET
case 8	F	16	tectal	ANET
case 9	F	13	posterior fossa	RGNT
case 10	F	14	hypothalamic and medullary metastases	RGNT
case 11	F	19	intraventricular (V4)	RGNT
case 12	F	14	cerebellar	RGNT
case 13	M	13	intraventricular (V4)	RGNT
case 14	M	14	pineal	PRP
case 15	M	12	pineal	PRP
case 16	F	6	3rd ventricle	PE
case 17	F	15	parietal	astroblastoma
case 18	F	17	intraventricular	neurcytoma
case 19	M	15	intraventricular	neurocytoma

*ANET* angiocentric neuroepithelial tumor, *F* female, *M* male, *PE* papillary ependymoma, *PRP* papillary tumor of the pineal region, *RGNT* rosette-forming glioneuronal tumor of the fourth ventricle, *y* years

**Fig. 1:**
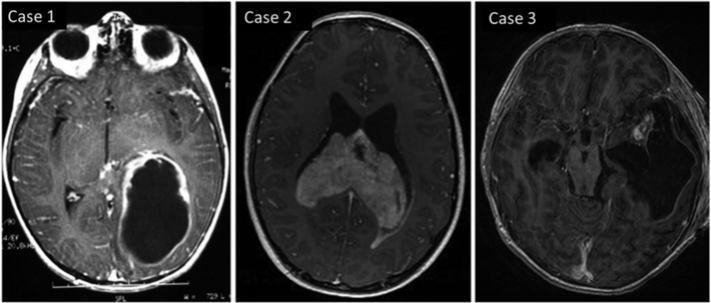
Axial T1-weighted pre-operative MR images for cases 1, 2 and 3 with nodular contrast enhancement and showing a ventricular association

We analyzed four pediatric patients with PGNT, two males and two females. Median age at diagnosis was 7 years (range, 5–14). Symptoms at diagnosis were epilepsy (cases 1 and 3) and intracranial hypertension (cases 2 and 3). In the case 4, the tumor has been revealed by an intracerebral hematoma. Complete resection was reported in cases 1, 2 and 3. One patient received adjuvant radiotherapy and presented a second location treated by surgery (case 1). The follow-up times were respectively 6 years, 9 years, 3 years and 9 months. At the time of the latest data, all patients were alive. Initial MRIs of three cases of PGNT (cases 1, 2, and 3) are shown in Fig. 1. No pre-operative imagery was available for case 4. In cases 1, 2 and 3, the tumors appeared as a well-demarcated tumor. All cases showed a ventricular association, one is intraventricular (case 2) and in the two others, the tumor had a contact with the temporal horn ventricle (case 1 and 3) and presented a cyst component. The three cases presented a contrast enhancing nodule.

### Histology

Histological characteristics are summarized in Table 3.

**Table 3:** Summary of histological and immunochemical characteristics

	Ganglioid cells	Granular bodies	GFAP	Synaptophysin	Chromogranin	CD34
case 1	-	-	+	+	+	-
case 2	-	-	+	+	+/-	-
case 3	-	+	+	+	-	+
case 4	-	-	+	+	+	+

**Fig. 2:**
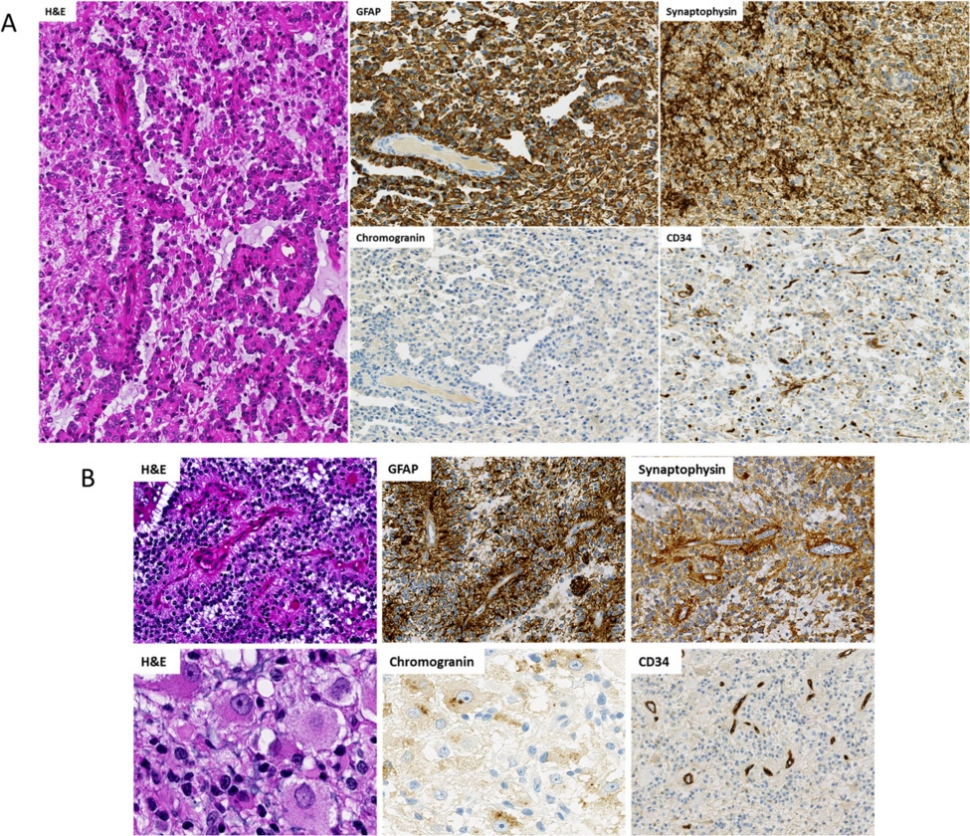
Histological and immunochemical characteristics. Serial sections from case 3 (**a**) showing a papillary architecture (H&E) with a GFAP positive glial component overlaying hyalinized vessels, synaptophysin positive interpapillary regions, absence of chromogranin positive cells but a CD34 extravascular expression. Serial sections from case 5 (**b**) showing a perivascular architecture and ganglion cells (H&E) with a GFAP positive glial component overlaying hyalinized vessels, synaptophysin positive interpapillary regions, with numerous chromogranin positive ganglion cells but an absence of CD34 extravascular expression. 2A, 2B GFAP, synaptophysin, CD34 magnification x100, 2B H&E, chromogranin magnification x200

All classical tumors classified as PGNT featured papillary and solid areas, were biphasic and characterized by a GFAP positive papillary glial component overlaying hyalinized vessels. Interpapillary regions were exclusively composed of homogeneous small cells, oligodendrocyte-like and neurocyte-like cells, chromogranin and/or synaptophysin positive and devoid of gangliocytoid and ganglioid cell features (Fig. 2a). We observed exceptional eosinophilic granular bodies in case 3 and hemorrhagic features in case 4. None of the four tumors showed malignant features. In all cases, Olig2-expressing oligodendrocyte-like cells were observed. An extravascular stellar expression of CD34 was only present in case 3. Into the PGNT mimics group, we took a particular attention in gangliogliomas with pseudopapillary features (cases 5 and 6). A ganglion cell component was observed with chromogranin positive ganglioid cells and binuclear cells. Abundant eosinophilic granular bodies and an extravascular stellar expression of CD34 were observed in both cases (Fig. 2b). Case 6 also harbored perivascular lymphoid infiltrates and hemorrhagic features.

### SLC44A1-PRKCA FISH analysis

**Fig. 3:**
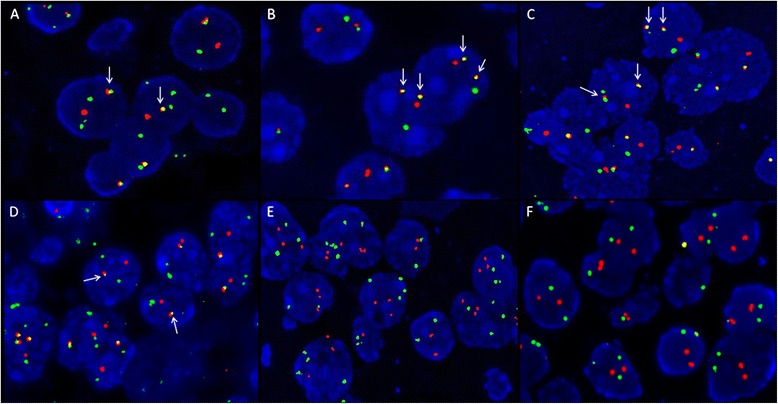
Interphase FISH analysis in PGNT cases using *SLC44A1* (green) and *PRKCA* (orange) dual-color probes (**a** and **d**) showing one *SLC44A1-PRKCA* fusion signal (white arrows) accompanied by one separate orange and two separate green signals in cases 1 and 4. **b** and **c** showing two *SLC44A1-PRKCA* fusion signals (white arrows) with one pair of separated orage and green signals in cases 2 and 3. **e** and **f** showing a lack of *SLC44A1-PRKCA* fusion signal in cases 5 and 6. Magnification x600

FISH analysis conducted on representative FFPE tissue sections of all PGNTs revealed a fusion of *SLC44A1-PRKCA* in a high percentage of the tumor nuclei examined, indicating a rearrangement of these loci. Specifically, in cases 1, 2, 3 and 4, fusion signals were respectively observed in 82, 93, 74 and 77 %, of the counted nuclei, (Fig. 3a-d). Among these tumors, two different profiles were identified; in cases 2 and 3 a majority of positive cells showed two fusion signals with one pair of separated orange and green signals (Fig. 3b and c). In cases 1 and 4, positive nuclei showed only one fused orange/green signal accompanied by one separate orange and two separate green signals (Fig. 3a and d).

In an effort to verify the specificity of the *SLC44A1*-*PRKCA* fusion, FISH analysis was also performed on fifteen PGNTs mimics cases (cases 5 to 19) and demonstrated that none of these cases displayed the *SLC44A1-PRKCA* fusion (Fig. 3e and f).

### Immunohistochemical and molecular findings

Molecular findings are summarized in Table 4.

**Table 4:** Immunohistochemical and molecular characteristics of PGNT cases

	*SLC44A1-PRKCA* (FISH)	*KIAA1549-BRAF* (RT-PCR)	*BRAF* mutation (IHC and/or sequencing)	*FGFR1* mutation (sequencing)	Phospho-ERK (IHC and/or WB)	H3.3 mutation (IHC)	p53 accumulation (IHC)
case 1	+	-	-	-	+	-	-
case 2	+	-	-	-	+	-	-
case 3	+	-	-	-	+	-	-
case 4	+	ND	-	-	+	-	-

*IHC* immunohistochemistry, *ND* not done, *WB* western blot

**Fig. 4:**
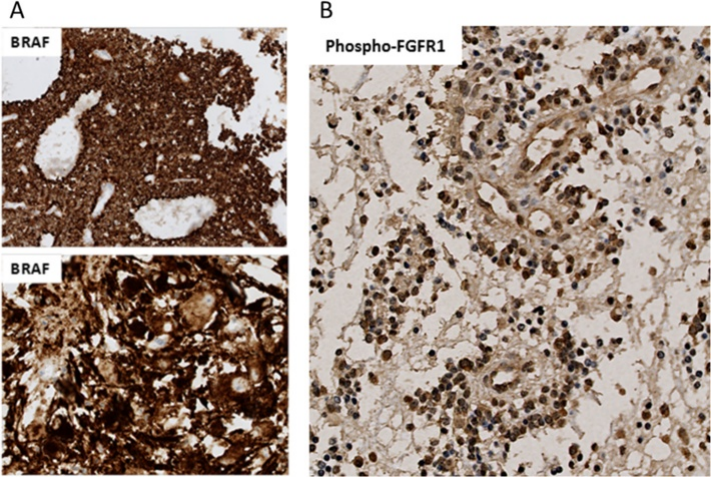
BRAF and phospho-FGFR1 immunostaining. **a** Immunohistochemistry on section from case 5 showing tumor cells stongly labeled in the two components with an antibody against BRAF V600E. **b** Immunohistochemistry from case 1 showing the tumor immunoreactive with an antibody against phospho-FGFR1. Magnification x200

We analyzed the nuclear accumulation of p53 and established the H3-K27 mutational status by immunohistochemistry; neoplastic cells were negative for both in all PGNTs. Interestingly, the two gangliogliomas with pseudopapillary features, which are negative for *SLC44A1*-*PRKCA* fusion, were *BRAF* mutation immunopositive (Fig. 4a). In case 5, the c.1799 T > A (p.V600E) *BRAF* mutation was confirmed by sequencing (but for case 6 DNA extraction was of poor quality and sequencing failed). Conversely, none of *SLC44A1*-*PRKCA* positive cases were positive for the *BRAF* V600E mutation on immunostains, except case 4. In case 4, the *BRAF* mutation was not confirmed by the sequencing analysis. Furthermore, we looked for *KIAA1549*-*BRAF* fusion by RT-PCR on cases 1, 2, and 3. None of those tumors showed the fusion. On immunostainings, the cases 1, 2 and 3 showed a weak phospho-FGFR1 expression (Fig. 4b) and the case 4 was negative. In case 3, the labeling was present exclusively in the papillary component while in the other it was diffusely present. None of the PGNTs were positive for *FGFR1* mutation by sequencing. Exon 12 could not be analyzed in cases 1 et 2.

### Phospho-ERK analysis

**Fig. 5:**
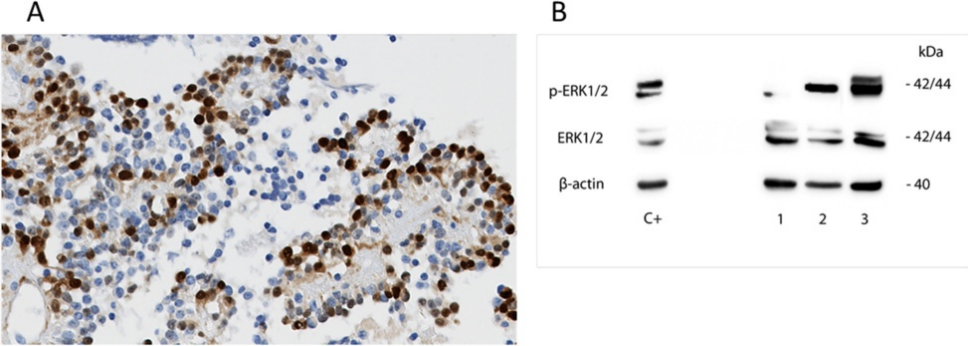
Phospho-ERK analysis. **a** Immunohistochemistry on section from case 1 showing a strong expression of phospho-ERK. Magnification x200. **b** Total lysates from individual tumors (case 1, 2 and 3) were subjected to western blotting for expression analyses of phosphorylated (p-) and non-phosphorylated ERK1/2. Pilocytic astrocytoma expressing *KIAA1549-BRAF* fusion was used as positive control (C+). ß-actin was used as reference

In all PGNTs, the tumor cells and particularly the papillary components were strongly labeled with phospho-ERK antibodies (Fig. 5a). These findings were confirmed by western blotting (Fig. 5b) in all three cases analyzed (cases 1, 2, 3), indicating an activation of the MAPK-signaling pathway.

## Discussion

PGNT is a rare mixed glioneuronal tumor usually characterized by an indolent clinical behavior [21]. The rarity and the scarcity of tissue available for molecular and genetic analyses have hampered the identification of specific alterations. The diagnosis of PGNT is often difficult to establish both on MRI and histology. On MRI, PGNT is frequently described as a well-demarcated mass with a cystic component, contrast enhancement, isointensity on T1-weighted and a predominant location in the periventricular area [22]. However, there is no pathognomonic radiographic appearance and in the review by Carangelo and colleagues, around 80 % of PGNT showed a ventricular association [3]. All our cases show a ventricular association, one is clearly intraventricular, similar to the case reported by Matyja and colleagues [23] and the two others, the tumor have a direct contact with the temporal ventricle. The three cases present a nodular contrast enhancement. Cases 1 and 3 show a more typical appearance with a cystic component. According to the WHO 2007 classification, PGNT is histologically characterized by a distinct pseudopapillary architecture with layers of GFAP positive glial elements, lining hyalinized vessels and variable amount of interpseudopapillary spaces filling by synaptophysin-positive neurocyte-like, ganglioid, or ganglion cells [2]. However, microcystic oligodendroglial-like areas and astrocytic components could resemble to pilocytic astrocytoma or ANET. The diagnosis is thus based on a set of histological and radiological criteria. However, individual histological entities could classically overlap with other LGG entities (PGNT mimics). In particular, the distinction between PGNT and ganglioglioma is not yet completely clear as in WHO description, PGNT could contain a heterogeneously distributed ganglion cell tumoral population [2]. Bridge and colleagues identified a recurrent chromosomal translocation t(9;17)(q31;q24) in three PGNTs resulting in an in-frame fusion of *SLC44A1* and *PRKCA*, with consequent generation of a constitutively expressed serine/threonine kinase fusion [12]. A recent study has reported the presence of the fusion in two additional PGNTs [13]. Consistent with this, our findings confirm the presence of *SLC44A1*-*PRKCA* fusion in PGNT. We took a particular attention in the group of PGNT mimics of two particular cases of ganglioglioma with pseudopapillary features (cases 5 and 6). Indeed, histological features of this entity are similar to those of PGNT. However, interestingly, the cases 5 and 6 did not show the *SLC44A1-PRKCA* fusion but were *BRAF* V600E mutation positive, as described in around 40 % of gangliogliomas [24]. Ganglioglioma is also composed of neoplastic mature ganglion cells in combination with neoplastic glial cells [25]. In our cases, a ganglion cell component was observed only in cases 5 and 6 associated with eosinophilic granular bodies. Conversely, positive cases for *SLC44A1*-*PRKCA* fusion consisted exclusively of small oligodendrocyte-like cells and neurocyte-like cells without gangliocytoid and ganglioid cell features. Furthermore, case 6 showed an extravascular expression of CD34, a marker consistently expressed in 70–80 % of gangliogliomas, especially those variants emerging from temporal lobe [26]. The neoplastic cells from case 5, located in chiasmatic region, did not express CD34. All these findings argue for definitively classifying these two tumors as papillary variant form of gangliogliomas, and to clearly separate them from PGNT. Notably, it could be interesting as ganglioglioma represent the single glio-neuronal tumoral entity susceptible to spontaneously present a malignant transformation [2]. Indeed, relatively little is known about malignant changes in PGNT [27]. Several cases with high proliferative activity have been reported but all showed malignant features at diagnosis and to our knowledge, no secondary malignant transformation has been described in PGNT [28, 29]. Besides, molecular data concerning malignant PGNT are very limited; *BRAF* V600E mutation was screened in two cases which were both negative [6]. Recurrent somatics alterations of *FGFR1* are reported in pLGG [10] but no large pLGG cohort studying *FGFR1* mutational status have included some PGNT. Contrary to the single case report describing a *FGFR1* N546K mutation (located in the exon 14) [11], we did not find *FGFR1* mutation in our cohort. No correlation seems to exist between the presence of a mutation and a positive immunostaining using phospho-FGFR1 antibody.

The identification of *SLC44A1*-*PRKCA* may also be useful to distinguish PGNT from other tumors with perivascular/angiocentric features.

In order to shed more light on the specificity of *SLC44A1*-*PRKCA* fusion and its potential value in the differential diagnosis of PGNT, we also investigated thirteen additional cases of PGNT mimics. Particularly, we studied five RGNTs cases that could be a differential diagnosis due to the periventricular location and the biphasic pattern. Two RGNTs cases have been previously tested for *SLC44A1-PRKCA* fusion and were negative [13]. In our cohort, none of ANETs, RGNTs, PRPs, PE, astroblastoma, neurocytomas studied showed the *SLC44A1-PRKCA* fusion, further supporting PGNT as a unique entity. PRKCA is the alpha isoform of protein kinase C, a family member of serine and threonine kinases that can be activated by calcium and the second messenger diacylglycerol. PRKCA can promote cell growth by phosphorylating and activating RAF1, which mediates the activation of the MAPK signaling pathway. Therefore, deregulation or high constitutive level of *PRKCA* expression secondary to fusion with *SLC44A1* could directly result in the deregulation of the MAPK signaling pathway as observed in tumors showing *BRAF* V600E mutation [30], notably gangliogliomas, or *KIAA1549*-*BRAF* fusion as in pilocytic astrocytomas (PA) [8]. In our cohort, phospho-ERK analysis provides arguments for the activation of the MAPK signaling pathway.

MAPK pathway and *BRAF* status have been widely explored over the last few years showing that the histologic spectrum of low grade gliomas and glioneuronal tumors is vague but individualizing a group of low grade glioma with MAPK signaling pathway deregulation. PGNT is a rare entity which belongs to this group with MAPK signaling pathway deregulation. *SLC44A1*-*PRKCA* fusion alone could drive tumorigenesis, but additional studies are needed to confirm this hypothesis.

## Conclusions

In conclusion, in this study including clinico-radiological and follow-up data, we have confirmed and extended the molecular data on pediatric PGNT. We uphold the presence of *SLC44A1*-*PRKCA* fusion in PGNT, which could represent a novel model of single pathway disease as PA. *SLC44A1*-*PRKCA* fusion seems to be specific for PGNT with a high diagnostic value. As a consequence, we recommend investigating it by FISH analysis for a diagnosis confirmation.
